# Long-Term Impacts of Defoliator Outbreaks on Larch Xylem Structure and Tree-Ring Biomass

**DOI:** 10.3389/fpls.2020.01078

**Published:** 2020-07-15

**Authors:** Daniele Castagneri, Angela L. Prendin, Richard L. Peters, Marco Carrer, Georg von Arx, Patrick Fonti

**Affiliations:** ^1^Swiss Federal Institute for Forest, Snow and Landscape Research WSL, Birmensdorf, Switzerland; ^2^Department TeSAF, Università degli Studi di Padova, Padova, Italy; ^3^Laboratory of Plant Ecology, Ghent University, Ghent, Belgium

**Keywords:** cell wall, defoliation, insect outbreak, *Larix decidua*, quantitative wood anatomy, tracheid, xylem functional traits, wood biomass

## Abstract

Defoliator insects are a major disturbance agent in many forests worldwide. During outbreaks, they can strongly reduce photosynthetic carbon uptake and impact tree growth. In the Alps, larch budmoth (*Zeiraphera diniana*) outbreaks affect European larch (*Larix decidua*) radial growth over several years. However, immediate and legacy effects on xylem formation, structure, and functionality are still largely unknown. In this study, we aimed at assessing the impact of budmoth defoliations on larch xylem anatomical features and tree-ring structure. Analyses were performed in the Lötschental (Swiss Alps) within (1,900 m a.s.l.) and above (2,200 m a.s.l.) the optimum elevational range of larch budmoth. We investigated variability of xylem anatomical traits along century-long tree-ring series of larch (host) and Norway spruce (non-host) trees. We identified eight outbreaks affecting larch xylem anatomy during the 20^th^ century, particularly at 1,900 m a.s.l. Tracheid number always showed a higher percent reduction than properties of individual cells. Cell lumen size was slightly reduced in the first 2–3 years of outbreaks, especially in the early part of the ring. The more carbon-demanding cell wall was thinned along the entire ring, but more evidently in the last part. Theoretical tree-ring hydraulic conductivity was reduced for several years (up to 6), mostly due to cell number decrease. Reduced cell wall area and cell number resulted in a strong reduction of the tree-ring biomass, especially in the first year of outbreak. Our study shows that, under carbon source limitations caused by natural defoliation, cell division is more impacted than wall thickening and cell enlargement (the least affected process). Consequences on both xylem hydraulic properties and tree-ring biomass should be considered when assessing long-term defoliator effects on xylem functioning, forest dynamics, and terrestrial carbon cycle.

## Introduction

During their long lifespan, trees are exposed to climate variability, stand dynamics (inter-tree interactions), and biotic and abiotic disturbances, which strongly influence physiological processes, including radial stem growth (Cook, 1987). Much research has focused on the influence of temperature and precipitation on inter-annual tree growth variability (Babst et al., 2019). Still, large-scale natural disturbances are an integral part of forest dynamics (Seidl et al., 2017) and can strongly affect growth processes.

Insect outbreaks are a major disturbance in temperate and boreal forests, significantly affecting nutrient cycling, forest productivity, carbon sequestration, and biodiversity (Kautz et al., 2017). In Europe, the most relevant biotic disturbance agents are bark beetles (Schelhass et al., 2003). However, defoliators can also cause severe damages at regional scale (Netherer and Schopf, 2010; Foster, 2017). One of the most studied species is *Zeiraphera diniana* Guenée (= *Zeiraphera griseana* Hübner, larch budmoth, hereafter LBM) that causes large defoliations on European larch (*Larix decidua* Mill.), a conifer widespread in the European Alps. Past studies showed that larch is highly sensitive to temperature variability, which influences leaf phenology (Busetto et al., 2010), cone production (Poncet et al., 2009), radial stem growth rates (Carrer and Urbinati, 2006), xylem phenology (Cuny et al., 2019) and structure (Carrer et al., 2017). However, the role of LBM should also be considered as severe outbreaks can deeply reduce carbon assimilation, causing strong growth reduction for several years (Peters et al., 2017). Defoliations mostly occur between 1,700 and 2,000 m a.s.l., last for one to three years, and typically occur every 8–10 years (Baltensweiler and Rubli, 1999; Baltensweiler et al., 2008; Büntgen et al., 2009; Büntgen et al., 2020). However, the last 40 years witnessed only very moderate periodic defoliations (Johnson et al., 2010; Wermelinger et al., 2018). Several studies have documented the effects of LBM on the width, density, and stable isotope composition of tree rings from different Alpine regions (Nola et al., 2006; Kress et al., 2009; Weidner et al., 2010; Battipaglia et al., 2014; Hartl-Meier et al., 2017; Cerrato et al., 2019). Despite these numerous studies, there is still a lack of knowledge on how LBM affects xylem structure. Retrospective analysis of a number of xylem anatomical traits can be used therefore to gain further insight into the impact of outbreaks on stem functioning and the amount of structural carbon stored in wood (Locosselli and Buckeridg, 2017). Among them, the size of the cell lumen, besides other features such as pit structure (Choat et al., 2008), is critical for water transport, as hydraulic conductivity depends on the fourth power of the conduit diameter (Hagen–Poiseuille law). Cell wall thickness in the latewood defines latewood density (Björklund et al., 2017), which confers mechanical support (Chave et al., 2009), while thick walls in the earlywood prevent cell implosions caused by negative xylem pressures (Rosner et al., 2016). Integrating information from all the cells in the ring, it is possible to assess the structure and potential functioning of the entire tree ring. The sum of theoretical hydraulic conductivity of all the cells defines the theoretical tree-ring hydraulic conductivity (Tyree and Zimmermann, 2002; von Arx and Carrer, 2014). Summing the cell wall area (Fonti et al., 2013) of all tracheids provides an estimate of wood material in the ring (tree-ring biomass) (Björklund et al., 2019). Finally, the ‘hydraulic carbon use efficiency’ expresses the (yearly) theoretical tree-ring hydraulic conductivity for a given carbon investment (Prendin et al., 2018).

So far, a handful of studies has investigated defoliator effects on conifer xylem anatomical traits. These studies often only assessed single responses (generally reductions) of tracheid number (Krause and Morin, 1995), lumen size (Filion and Cournoyer, 1995; Camarero et al., 2019), and latewood cell wall thickness (Axelson et al., 2014; Paixao et al., 2019), of just one event, providing a partial view of the species-specific xylem response to outbreaks. In this study, we inspected radial growth patterns and several xylem functional traits for a more integrative and deeper understanding of European larch responses to LBM defoliations. Sampling was conducted in the Lötschental valley, Switzerland, within (1,900 m a.s.l.) and above (2,200 m a.s.l.) the optimum elevational range of the insect. Retrospective analysis on tree-ring series covered the entire 20^th^ century. We examined cell anatomical parameters at the intra-ring level, to evaluate modifications of cells formed in different periods of the growing season. In addition, we assessed LBM impacts on the theoretical tree-ring hydraulic conductivity and the tree-ring biomass.

We hypothesized that: (1) lumen size, mostly dependent on turgor pressure during cell enlargement (Taiz and Zeiger, 2006; Cabon et al., 2020a), and the derived cell and tree-ring theoretical hydraulic conductivity, would be not or only slightly affected by outbreaks; on the contrary (2) cell-wall thickness and cell number, directly related to carbon availability (Cuny et al., 2015; Cartenì et al., 2018), would be strongly negatively affected. We tested the stability of these patterns during episodes of different outbreak severity, within and above the LBM optimum. Furthermore, we evaluated whether the relative reduction in tree-ring width during outbreaks differed from the relative reduction in tree-ring biomass estimated from xylem anatomy. We hypothesized (3) that the estimate from xylem anatomy, considering variations of tracheid size, could provide significantly different and more accurate estimate of biomass loss in tree rings compared to tree-ring width.

## Materials and Methods

### Sample Collection

The study was conducted at the southeast-facing slope of the Lötschental valley, in the central Swiss Alps (46°23′40″N, 7°45′35″E), where previous dendrochronological studies evidenced several LBM outbreaks in the 20^th^ century (Esper et al., 2007; Kress et al., 2009; Peters et al., 2017). The approx. 15 km near weather station of Crans-Montana (1,427 m a.s.l., 1931–2018) has registered a mean annual temperature of 5.8°C and a mean annual precipitation sum of 925 mm. The slope is covered by a forest mostly composed of European larch and Norway spruce (*Picea abies* (L.) Karst.). Two sample sites have been selected. One was located within the optimum elevational range of LBM at 1,900 m a.s.l. (S19), where we expected stronger outbreak effects on larch xylem anatomy, and the second one above the optimum, at the forest elevational limit (2,200 m a.s.l., S22). Besides larch, we also sampled non-host Norway spruce at S19, to disentangle the effects of species-specific causes (LBM outbreak) from other large-scale factors that could affect growth of all species, such as climate (Swetnam and Lynch, 1993).

At each site, one increment core from 12 adult (supposedly > 100 years), not suppressed, and undamaged trees was collected at 1.3 m above ground, on the uphill side of the stem to minimize the influence of compression wood (Weber, 1997). Tree-ring widths were measured to the nearest 0.01 mm using TsapWin (Rinntech, Heidelberg, Germany), and cross-dating quality was checked using COFECHA (Holmes, 1983). Cell anatomical analyses were performed on a selection of seven cores from larch at each site and seven spruce at S19 (see **Supplementary Table S1** for summary statistics), avoiding cores with nodes, reaction wood, or with rotten or missing parts.

### Anatomical Measurements

The cores were cut into 4–5 cm long pieces to prepare transversal sections with a rotary microtome (RM2245, Leica, Heidelberg, Germany). The sections were stained with safranin (1% in distilled water), permanently fixed on the slides and finally scanned at 100× magnification using a digital automated microscope (D-sight, Menarini Diagnostic, Florence, Italy, and Axio Scan.Z1, Zeiss, Jena, Germany; Gärtner et al., 2015; von Arx et al., 2016). Tracheid anatomical measurements were performed using ROXAS software (v. 3.1, von Arx and Carrer, 2014; Prendin et al., 2017) that depends on the commercial software Image-Pro Plus v. 6.1 (Media Cybernetics, Rockville, MD, USA).

**Figure 1:**
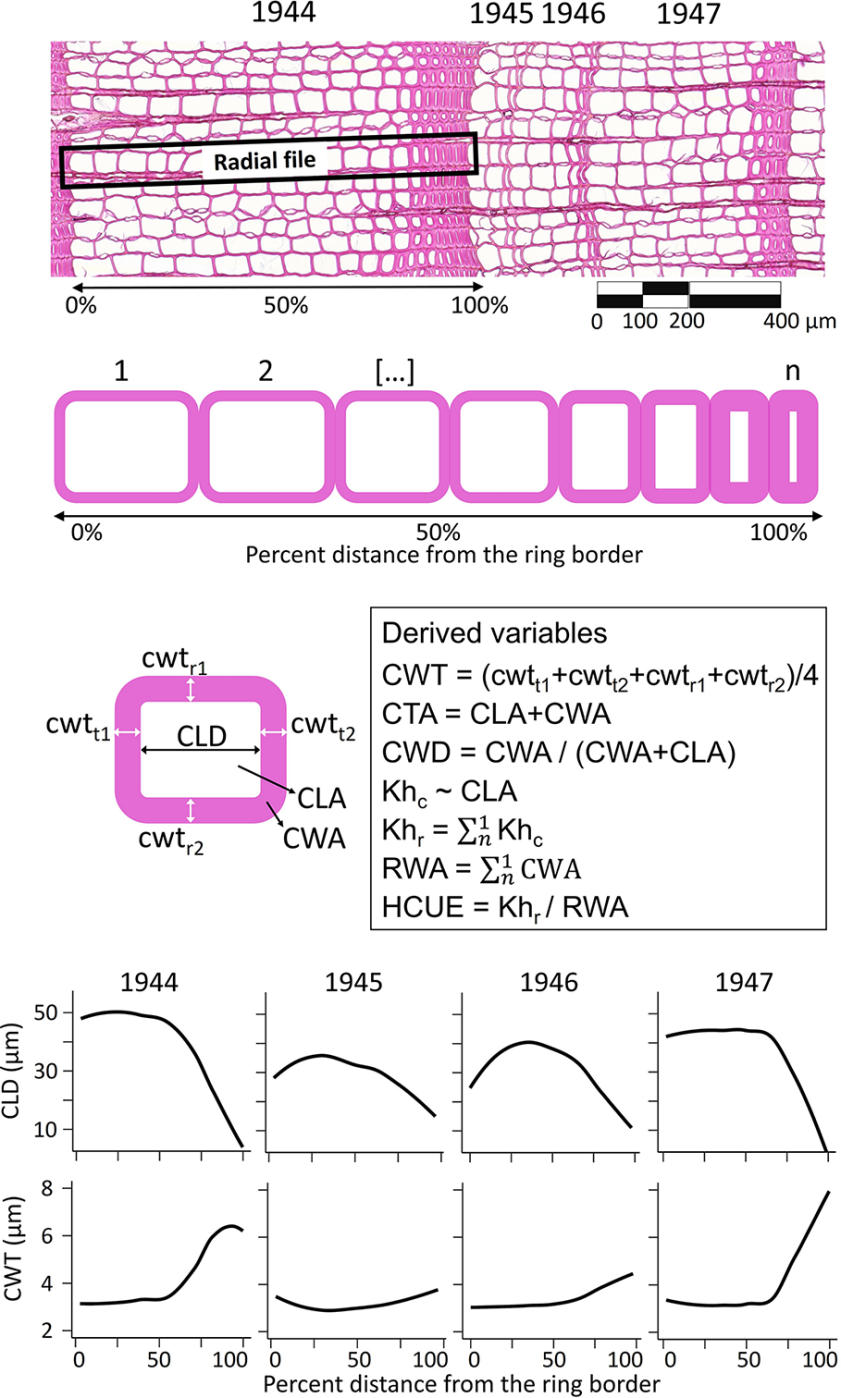
Illustration of anatomical data extraction and computation. Within each ring and along each radial file, for every tracheid (from 1 to n, where n is the cell number, CN) we measured the cell lumen radial diameter (CLD), mean cell-wall thickness (CWT), cell lumen area (CLA), cell-wall area (CWA), cell total area (CTA), relative anatomical cell wood density (CWD), and theoretical cell hydraulic conductivity (Kh_c_). From this information, we derived intra-ring profiles (*i.e.* loess fit of the anatomical parameter for each cell and its relative position in the ring, lower panels) and ring-based traits as theoretical tree-ring hydraulic conductivity (Kh_r_), ring wall area (RWA), and hydraulic carbon use efficiency (HCUE). Anatomical data used in this figure come from rings of a larch tree at S19, before (1944) and during the high-severity outbreak starting in 1945.

Anatomical measurements were performed for each annual ring in the common period 1900–2017 (**Supplementary Table S1**). For each ring, we obtained the ring width (RW). For each tracheid, we characterized its position within the ring, and we measured the radial cell lumen diameter (CLD), mean cell-wall thickness (CWT, as the average of radial and tangential wall thickness), cell lumen area (CLA), cell-wall area (CWA), total cell area (CTA, as CLA + CWA), theoretical cell hydraulic conductivity (Kh_c_) approximated according to the Poiseuille’s law and adjusted to elliptical tubes (Tyree and Zimmermann, 2002), and relative anatomical cell wood density (CWD, as CWA/(CWA+CLA); **Figure 1**, **Supplementary Table S1**). We then used the RAPTOR R package (Peters et al., 2018) to assign tracheid to radial files and obtain the mean number of cells per radial file (CN). This information provided the basis to calculate the ring wall area (RWA) as the sum of all CWA of the average radial file. Considering that >90% of conifer wood is typically formed of tracheids (Carlquist, 1988; Chave et al., 2009), RWA was a reliable estimate of the biomass in the tree ring (Björklund et al., 2019). Besides, since wood carbon content in conifer cell wall is quite uniform (50.8 ± 0.7% [95% C.I.], Thomas and Martin, 2012), RWA was also a quite precise estimate of the amount of structural carbon in the ring. The theoretical tree-ring hydraulic conductivity (Kh_r_) was assessed as the sum of Kh_c_ of the average radial file (**Figure 1**). The ratio between Kh_r_ and RWA provided the hydraulic carbon use efficiency (HCUE; Prendin et al., 2018).

### Assessing LBM Outbreak Impacts on Xylem Anatomy

**Figure 2:**
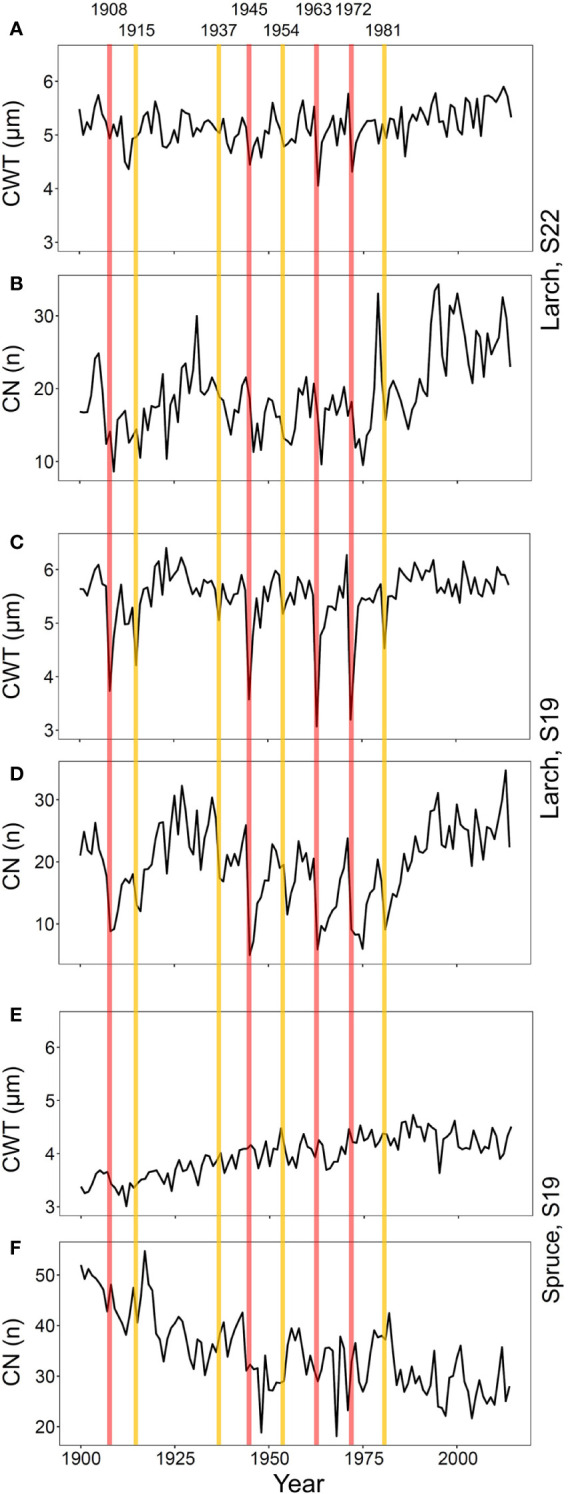
Chronologies of mean-ring cell-wall thickness (CWT, **A**, **C**, **E**) and cell number (CN, **B**, **D**, **F**) from 1900 to 2017 for larch at S22 **(A**, **B)** and S19 **(C**, **D)** and spruce at S19 **(E**, **F)**. Vertical lines represent the first years of high- (red) and low- (yellow) severity outbreaks.

To verify correspondence with the 20^th^ century outbreaks reported in previous studies in this area (Esper et al., 2007; Kress et al., 2009), we investigated CN and CWT chronologies of larch and spruce (**Figure 2** and **Supplementary Figure S1**). In conifers, CN is strongly related to RW (Castagneri et al., 2015), which is the most commonly used parameter to detect LBM outbreaks (Büntgen et al., 2009). CWT mostly determines the tree-ring maximum wood density (Björklund et al., 2017), a proxy successfully used in past studies (Esper et al., 2007).

Since we expected that duration and effects of different outbreaks were highly variable as reported in previous studies (Weber, 1997), we separated the detected events in two equal-size classes. Based on CWT reductions at S19, where the outbreak effects were more evident, we distinguished: four high-severity outbreaks with marked CWT decline (with the strongest CWT reduction compared to previous five year reference period); and four low-severity ones with less marked CWT reduction (the other four identified events; **Figure 2**, **Supplementary Figure S1** and **Supplementary Table S2**). The same classification was also applied to S22 to compare anomalies at this site to those at S19 in the same years.

To quantify the outbreak effects on xylem structure, we calculated the deviation (ratio) of each parameter in the first year of the outbreak, from the mean in the previous five, considered as reference (Peters et al., 2017). Welch’s t-test was used to test for significant differences. The analysis was repeated for the successive seven years by keeping the five years prior to the outbreak as the reference. We then averaged this information for severity level and site.

Similarly, to assess the effects of outbreaks at the intra-ring level, we computed the intra-ring variation (profiles) of the anatomical parameters calculated at the cell level (*i.e.* CLD, CWT, CLA, CWA, CTA, Kh_c_, CWD) for every year (mean of seven trees) in the period 1900–2017 by considering high- and low-severity outbreaks. Therefore, the anatomical profiles per severity level and site were superposed to calculate cell anatomy variations in the averaged five years before the outbreaks (to serve as a reference) and in the individual successive years.

### Climate Influence on Larch and Spruce Anatomy

To verify whether xylem anatomy anomalies in larch were caused by LBM outbreaks, we investigated the responses in the non-host spruce. The comparative approach assumes that two species have similar responses to climate, thus anomalies in one species (larch in our case) can be attributed to species-specific causes (Swetnam and Lynch, 1993). To validate this assumption, we assessed climate responses of the two parameters used to detect outbreaks, CN and CWT (the latter split in earlywood and latewood, separated according to a Mork’s index of 1, Denne, 1989), in larch and spruce. Long-term trends, mostly due to tree radial and height growth during ontogenesis (Carrer et al., 2015), were removed by fitting a cubic smoothing spline with 50% frequency cut-off of 100 years. We then calculated the ratio between the observed and fitted curves (Cook and Kairiukstis, 1990) using the R package dplR (Bunn, 2008). Mean chronologies for larch at S22 and larch and spruce at S19 were built by calculating the bi-weight robust mean from the detrended series. We then calculated bootstrap correlations between each chronology and monthly temperature and precipitation from April to October (including the xylem growing season of larch in the area, Cuny et al., 2019) for the period 1931 to 2017 (covered by climate data from the Crans-Montana weather station) using the R package treeclim (Zang and Biondi, 2015). Correlations were calculated by both including and excluding outbreak years (Weidner et al., 2010).

## Results

Correlations between anatomical chronologies and monthly temperature and precipitation showed quite similar responses in larch at the two study sites (**Supplementary Figure S2**). High temperatures from May to August were related to high CN, and CWT in latewood was positively associated with temperature during early and, mostly, late summer. In spruce, CN was not affected by climate variability. In contrast, latewood CWT was positively associated with summer temperature, as for larch. That is, concomitant low CWT values in larch and spruce should be related to low summer temperature. CWT reductions during specific years in larch, not evidenced in spruce, were instead related to species-specific causes such as LBM outbreak.

Eight out of nine outbreaks reported in previous studies in this area (years 1908, 1915, 1935, 1945, 1954, 1963, 1972, and 1981; Esper et al., 2007; Kress et al., 2009) were evident in the CWT larch chronology at S19 (**Figure 2** and **Supplementary Figure S1**). The CN chronology, strongly correlated with RW chronology (Pearson’s correlation, r = 0.98, p < 0.001), was less accurate than CWT for detecting the outbreak start. The 1923 outbreak (described as weak in Weidner et al., 2010 and absent in Arbellay et al., 2018) was not noticeable in our samples as it did not show the CWT reduction typical for all other outbreak years.

**Figure 3:**
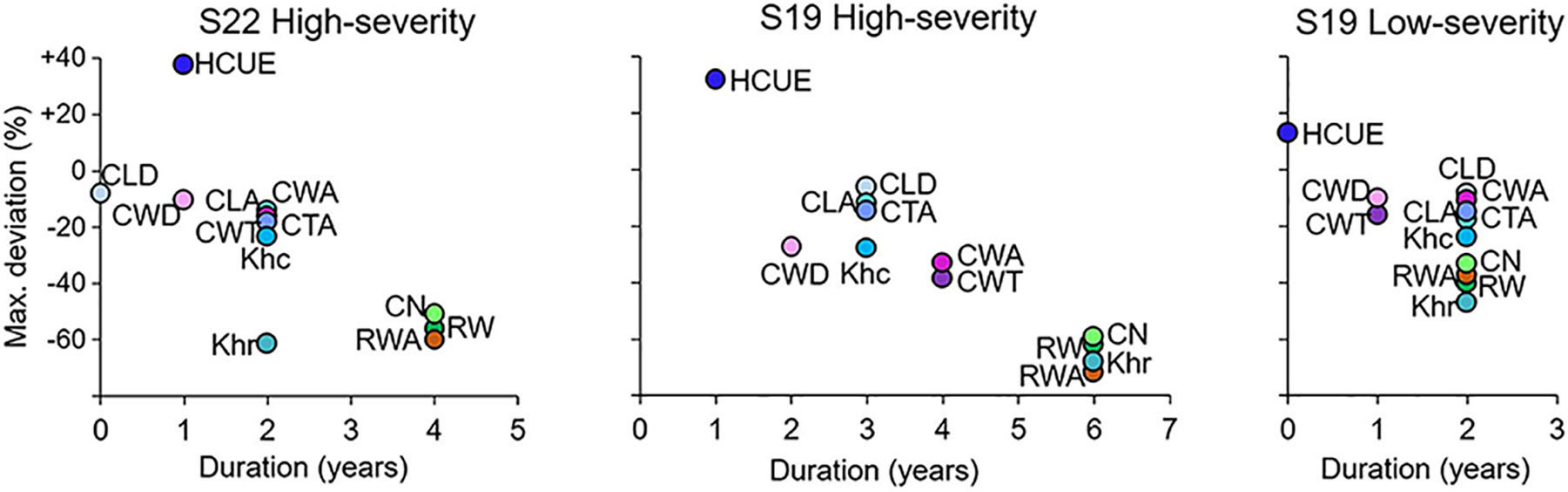
Scatterplot of the duration and maximum deviation of LBM outbreaks on larch xylem anatomical parameters. Parameters are: mean cell lumen radial diameter (CLD), mean cell-wall thickness (CWT), mean cell lumen area (CLA), mean cell-wall area (CWA), mean cell total area (CTA), mean theoretical hydraulic cell conductivity (Kh_c_), mean relative anatomical cell wood density (CWD), ring wall area (RWA), theoretical tree-ring hydraulic conductivity (Kh_r_), hydraulic carbon use efficiency (HCUE), ring cell number (CN), and ring width (RW). Maximum deviation is the percentage variation of the mean value of the parameter from the reference, *i.e.* the mean value in the five years before the outbreak, during the year of maximum reduction for an outbreak event. Duration corresponds to the number of years when the parameter was statistically different from the reference, according to Welch’s t-test. Deviations and durations are merged for the four high-severity and the four low-severity outbreaks, and for S19 and S22, separately. Parameters for low-severity years for larch at S22 are not presented since deviations were not significant.

The effects of the 1908, 1945, 1963, and 1972 outbreaks were more intense and lasted longer at S19 than at S22 (**Figures 2** and **3**, **Supplementary Figure S3**). At both sites, traits related to cell wall dimension (CWT, CWA, CWD) were more reduced than those related to lumen size (CLD, CLA, CTA, Kh_c_), especially in the first year. RW, CN, Kh_r_, and RWA were highly affected, both in the first and in the successive years. Xylem anatomical variations at S19 for low-severity outbreaks were similar to those at S22 during the 1908, 1945, 1963, and 1972 outbreaks. Anatomical parameters during the 1915, 1937, 1954, and 1981 outbreaks for larch at S22 and for all outbreak years for spruce at S19 were not significantly different from the reference; therefore they were not further investigated (**Supplementary Table S2**, **Supplementary Figures S3** and **S4**).

**Figure 4:**
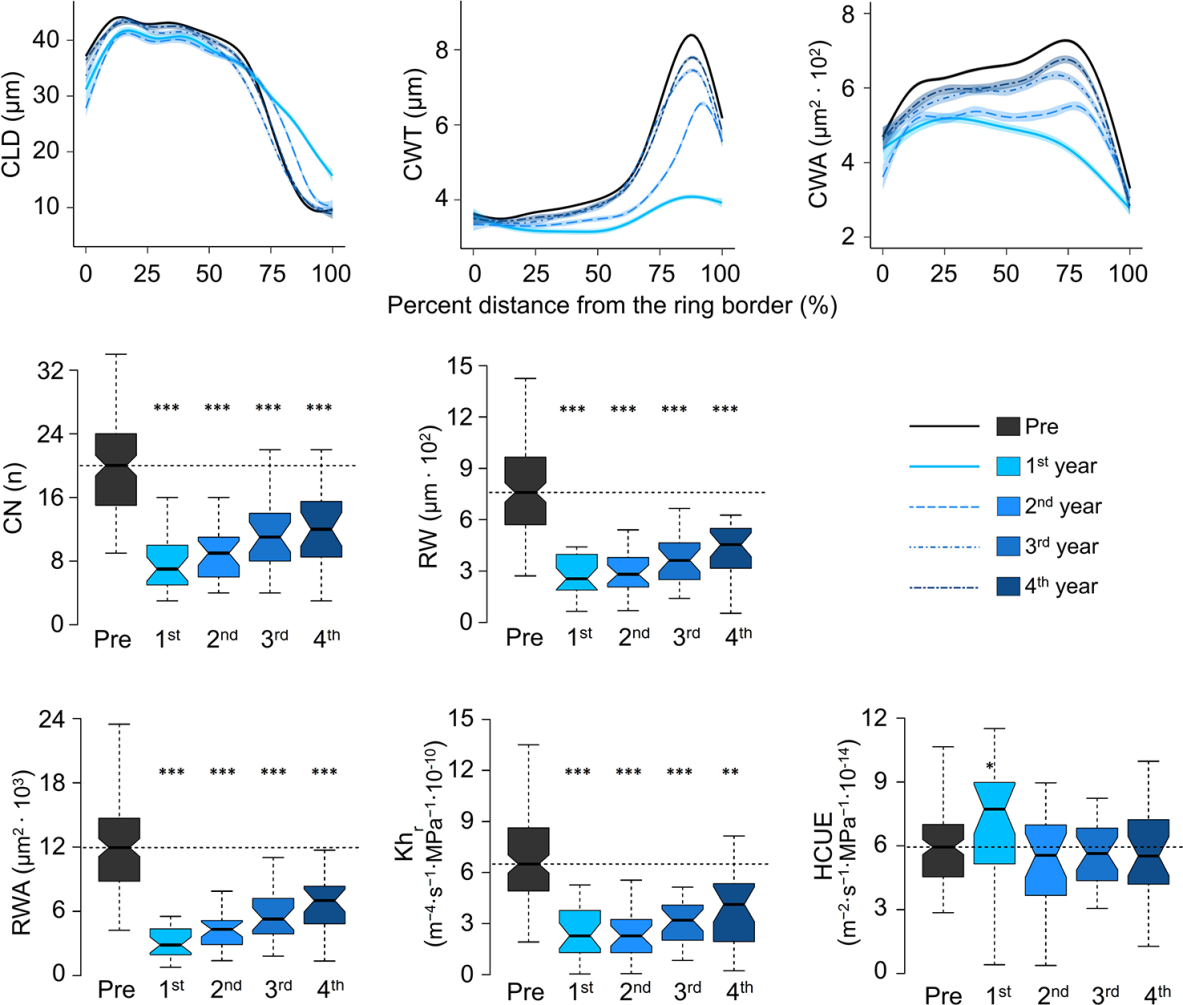
Evolution of cell anatomical characteristics around the outbreak events for the four high-severity outbreaks (started in 1908, 1945, 1963, and 1972) at S19. Pre = average of the five years before outbreak. 1^st^ is the year identified as outbreak start (see the key for correspondence between years and colors). CLD, cell lumen radial diameter; CWT, cell-wall thickness; CWA, cell wall area; CN, ring cell number; RW, ring width; Kh_r_, theoretical tree-ring hydraulic conductivity; RWA, ring wall area; HCUE, hydraulic carbon use efficiency. CLD, CWT, and CWA are standardised as percent distance from the ring border. The ring-specific profiles are loess fits, with standard error envelopes, of the anatomical parameter for each cell and its relative position in the ring. CN, RW, RWA, Kh_r_, and HCUE are calculated at the ring level and represented as box plots to indicate the median, interquartile range (IQR), confidence interval (notches, +/− 1.58·IQR/√n), minimum and maximum values. Dashed horizontal lines indicate the median value during “Pre”. Asterisks indicate significant difference from the reference (“Pre”) at p < 0.05 (*), p < 0.01 (**), and p < 0.001 (***) according to Welch’s t-test. Boxplots of all mean parameters are represented in **Supplementary Figure S3**. Profiles of all cell parameters are represented in **Supplementary Figure S4**.

Intra-ring profiles showed that the impact of outbreaks on xylem anatomy was not the same along the entire ring (**Figure 4**, **Supplementary Figure S4**). At S19, in the first year of the high-severity outbreaks, CLD was reduced in the first part of the ring. However, the lumen was slightly larger than usual in the last part of the ring. Such a pattern was still evident, but to a lesser extent, in the following year. CWT reduction in the first year of outbreak occurred along the entire ring, but it was more evident in the last part, normally characterized by very thick cell walls. In the second year, cell-wall thickness was still reduced, but the profile shape was more similar to the rings before the outbreak. At the fourth year, CWT was normal along most of the ring profile, but in the last part it was still thinner than the reference. Such alterations influenced the cell wall area (CWA), which was reduced along the entire profile, but more evidently in the last part. In the fourth year, CWA was still slightly smaller than before the outbreak. Similar intra-ring patterns were observed in the same years at S22 and for low-severity outbreaks at S19, but deviations from the reference profiles in the first year were less evident, and carry-over effects were shorter (**Supplementary Figure S4**).

**Figure 5:**
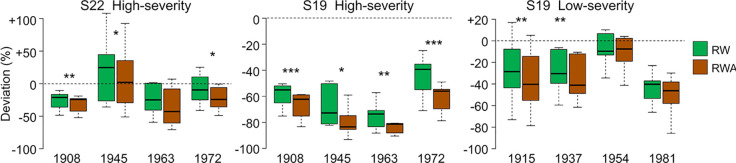
RW and RWA deviations from reference during the first year of outbreaks at S22 and S19. Deviation is the percentage variation of the parameter during the first year of each outbreak from the reference, *i.e.* the mean value in the five years before the outbreak. Each box represents median, interquartile range (IQR), minimum and maximum deviation from the reference. Asterisks indicate significant difference between RW and RWA deviation within the same year at p < 0.05 (*), p < 0.01 (**), and p < 0.001 (***), according to paired Welch’s t-test. Dashed lines indicate deviation equal to 0.

CN was highly reduced in the first year of high-severity outbreaks at S19. HCUE was the only tree-ring based parameter that showed significantly higher value than the reference in the first year. Instead, RWA and Kh_r_ were reduced by more than half, with significant deviations lasting for six years (**Figures 3** and **4**, **Supplementary Figure S3**). Although RW was significantly negatively affected, RWA reduction in the first year of most outbreaks was even stronger (**Figure 5**). Differences between RW and RWA were less evident in the successive years (**Supplementary Figure S4**). Reduction of CN, RWA, Kh_r_, and RW during low-severity outbreaks at S19 and high-severity outbreaks at S22 was highly significant but was less marked and lasted shorter than for high-severity outbreaks at S19.

## Discussion

### LBM Outbreak Effects on Cell Anatomical Features

Using a retrospective approach, we assessed the LBM long-term influence on larch xylem anatomy. We could ascribe observed xylem anomalies in larch to LBM outbreaks with high confidence. Although CWT of both larch (host) and spruce (non-host) was sensitive to summer temperature, CWT reduction during the eight detected outbreaks occurred just in larch (**Figure 2**). As expected, the outbreak effects were more evident at 1,900 than at 2,200 m a.s.l. (S19 and S22, respectively). Responses at S22 during the 1908, 1945, 1963, and 1972 outbreaks were quite similar to those at S19 during the 1915, 1937, 1954, and 1981 (low-severity) outbreaks. This suggests that factors other than climate variability (that is the same at the two close sites) influence LBM impact on xylem anatomy. Possibly, during heavy outbreaks, LBM can reach also high-elevation larches but with lower effects than on those within the insect elevational optimum.

Our analysis evidenced that LBM defoliation effects were not the same for all xylem functional traits. This indirectly indicates that xylogenesis phases of cambial division, cell enlargement, and cell wall thickening are differently affected not only by temperature and water (Castagneri et al., 2017; Cabon et al., 2020b), but also by carbon availability. Specifically, lumen size was reduced in the first two to three years of outbreak, but to a lesser extent than cell-wall thickness and cell number, confirming our initial hypothesis. Analysis at intra-ring level revealed that reduction in the first year was evident just in the first part of the ring, while in the last part, the lumen was larger than the reference (**Figure 4**). This could be due to reduced cell-wall thickness of the last cells, resulting in larger lumina. From anatomy, we could infer on the LBM effects on the processes that determine tracheid size. Cell enlargement is highly sensitive to water availability at the onset of growing season (Hsiao, 1973; Castagneri et al., 2017; Cabon et al., 2020a), whose variability is not related to LBM outbreaks. Still, reduced availability of carbon in the cambium could lower the osmotic potential and eventually impacts the cell enlargement rate (De Schepper and Steppe, 2010). Further, reduced carbon could affect primary wall synthesis during the enlargement phase (Cuny et al., 2015; Cartenì et al., 2018). Lumen size narrower than reference was evident even in the very first cells of the ring, indicating that LBM effects started very early in the season (cell enlargement of larch starts around early June at this site, Cuny et al., 2019). Interestingly, the typical cell-lumen size pattern within conifer rings was kept even during high-severity outbreaks. Despite the strong carbon shortage of high-severity outbreaks, both large earlywood and small latewood type-cells were formed. However, the last cells of the ring did not present the very thick walls typical of larch latewood. Thinner cell walls in the last part of the ring were also observed in other conifer species after defoliation (Liang et al., 1997; Axelson et al., 2014). However, intra-ring analysis evidenced that wall thickness was reduced not only in the tracheids formed at the end of the growing season, but along the entire ring, especially in the first year of high-severity outbreaks. Still, CWT never fell below ~3 µm (the wall thickness of earlywood cells during normal years), which probably represents a minimum biomechanical threshold for larch trees investigated here. Reduced CWT resulted in a noticeable decrease of the amount of wood material per cell (CWA). Cells formed in the first year of high-severity outbreaks at S19 comprised only about 2/3 of wood material compared to normal years. Such patterns agreed with our initial expectation. Secondary wall formation requires considerable carbon supply (Cuny et al., 2015; Deslauriers et al., 2015); therefore this process was heavily affected along the entire growing season during strong defoliations. After the first outbreak year, wall thickening was less affected, and the amount of wood material invested for each tracheid increased, yet for the next three years it was smaller than before the outbreak.

Compared to lumen size and cell-wall thickness, cell number was reduced more, and for more years. This was evident in both the study sites, and for both high- and low- severity outbreaks at S19. Defoliations reduced leaf photosynthetic capacity for some years, and caused long-term carbon reserve depletion (Baltensweiler et al., 2008). Six years after high-severity outbreak start at S19, cell number was still below pre-outbreak levels. Such reduction did not solely affect tree radial growth rates, as observed in previous studies on tree ring widths (Nola et al., 2006; Battipaglia et al., 2014; Peters et al., 2017), but also the tree-ring biomass, and its potential hydraulic functioning.

### LBM Outbreak Effects on the Theoretical Tree-Ring Hydraulic Conductivity and Tree-Ring Biomass

Analyses of tracheid anatomy revealed that lumen size was less reduced than wall thickness and cell number. However, the theoretical cell hydraulic conductivity (Kh_c_), which depends on the fourth power of conduit diameter (Tyree and Zimmermann, 2002), was much more reduced (25% less in the second year of high-severity outbreaks at S19) than cell lumen size. However, water transport in the stem depends not only on lumen size, but also on the number of conduits. The theoretical tree-ring hydraulic conductivity (Kh_r_), that represents the contribution of all tracheids formed in the year to the stem hydraulic system, was indeed more severely reduced (of 64% in the first year) than Kh_c_, due to the strong decrease of cell number. Even when lumen size returned to pre-outbreak values (*e.g.* after the third year, for high-severity outbreaks at S19), reduced cell number resulted in a loss of theoretical tree-ring hydraulic conductivity. The hydraulic carbon use efficiency (HCUE) evidenced that the balance between carbon used for building xylem (RWA) and the theoretical tree-ring hydraulic conductivity (Kh_r_) was quite stable despite large tracheid anatomical modifications. Such homeostasis was altered just in the first year of high-severity outbreaks when reduced carbon invested in xylem formation was counter-balanced by a more efficient xylem water transport.

Cell wall thinning certainly affected stem mechanical function. Despite a single ring with reduced wood density might not affect the entire tree stability, high-severity outbreaks reduced cell-wall thickness for four years, and many outbreaks occurred along the past century. Furthermore, reduced CWT resulted in a smaller cell wall area (CWA). This in turn affected the loss of tree-ring biomass during outbreaks. Previous studies quantified defoliator impacts on stem wood biomass or productivity through ring widths (Peters et al., 2017; Boyd et al., 2019; Paixao et al., 2019). However, these studies did not consider the xylem structure, which determines the wood density (Björklund et al., 2019). Missing such information can result in significant under/over estimations of wood biomass increments (Chave et al., 2009; Bouriaud et al., 2015; Pretzsch et al., 2018; Andrianantenaina et al., 2019). In the case of high severity outbreaks at S19, in the first year the estimate of biomass loss from ring width (classical approach) would be 26% lower (on average for the four outbreaks) than the assessment based on anatomical traits (RWA, considering both CN and CWA). From the second year, when CWT was less affected, estimates based on RW and RWA would converge.

## Conclusion

Periodic LBM defoliations strongly affected larch radial growth over the 20^th^ century in the investigated area. We here assessed the long-term impacts of outbreaks on cell anatomy and the tree-ring structure. This illustrated the effects of natural defoliations on xylem formation processes, potential functioning, and carbon accumulation in tree rings.

Both maximum impact (relative to the reference) and effect duration (number of years of significant reduction) of LBM outbreaks were stronger for tracheid number than for their anatomy, mostly confirming our initial hypotheses. This indicates that cambial division, that determines cell number, was more affected than the processes that shape the tracheid (anatomical) properties. Among the latter, cell wall thickening, which is a more demanding process in terms of carbon, was more affected than cell enlargement.

Over millennia of coevolution with LBM, larch evolved a strategy to primarily preserve traits related to water transport under strong carbon limitation. However, despite lumen size was the least affected trait, the theoretical tree-ring hydraulic conductivity was reduced for several years, due to the long carry-over effect of outbreaks on cell number. Reduced cambial division caused long-term radial growth reductions, but we demonstrated that even cell anatomy alterations (thinner cell-wall thickness, especially in the first year) significantly affected the amount of wood biomass accumulated in the tree rings. Our findings evidence that xylem anatomy should be considered in future assessments of defoliator impacts on tree physiology, forest dynamics, and terrestrial carbon cycle.

## Data Availability Statement

The data sets generated for this study are available on request to the corresponding author.

## Author Contributions

DC and PF conceived the study, with input from RP and GA. DC and AP performed laboratory and data analyses. DC performed the statistical analyses. DC drafted the paper, with contribution from AP, RP, MC, GA, and PF. All authors contributed to the article and approved the submitted version.

## Funding

DC received funding from the European Union’s Horizon 2020 research and innovation program under grant agreement No H2020-MSCA-IF-2017-788951. RP was supported by the Swiss National Science Foundation (SNSF), Grant P2BSP3_184475, AP by the 2017 BIRD Project of TeSAF Department University of Padova, and PF by the project LOTFOR-150205. Open access publication fees.

## Conflict of Interest

The authors declare that the research was conducted in the absence of any commercial or financial relationships that could be construed as a potential conflict of interest.

The handling Editor declared a past co-authorship with one of the authors PF.
