# Supplementary material for: Long-Term Impacts of Defoliator Outbreaks on Larch Xylem Structure and Tree-Ring Biomass
Source: Front Plant Sci. 2020 Jul 15;11:1078. doi: 10.3389/fpls.2020.01078 (PMC7378862; doi:10.3389/fpls.2020.01078)
Supplement: Supplementary file 1 [file DataSheet_1.docx]

**Supplementary material**

| Parameter | Unit |  | Larch S22 | | |  | Larch S19 | | |  | Spruce S19 | | |
| --- | --- | --- | --- | --- | --- | --- | --- | --- | --- | --- | --- | --- | --- |
| DBH | cm |  | 52.7 | (-6.5) | a |  | 51.5 | (-5.5) | a |  | 44.7 | (-3.1) | b |
| RN | n |  | 221 | (-51) | a |  | 189 | (-29) | b |  | 131 | (-31) | c |
| CLD | µm |  | 30.1 | (-1.8) | a |  | 29.3 | (-2.0) | b |  | 28.3 | (-1.3) | c |
| CWT | µm |  | 5.2 | (-0.37) | b |  | 5.52 | (-0.55) | a |  | 4.01 | (-0.43) | c |
| CLA | µm^2^ |  | 826 | (-84) | a |  | 814 | (-98) | a |  | 688 | (-56) | b |
| CWA | µm^2^ |  | 561 | (-41) | b |  | 574 | (-60) | a |  | 442 | (-43) | c |
| CTA | µm^2^ |  | 1266 | (-106) | a |  | 1286 | (-129) | a |  | 1087 | (-68) | b |
| Kh_c_ | m^4^·s^-1^·Mpa^-1^ |  | 3.73·10^-11^ | (5.84·10^-12^) | b |  | 3.91·10^-11^ | (7.74·10^-12^) | a |  | 2.37·10^-11^ | (3.41·10^-12^) | c |
| CWD |  |  | 0.56 | (-0.03) | b |  | 0.58 | (-0.04) | a |  | 0.48 | (-0.03) | c |
| RWA | µm^2^ |  | 11969 | (-4071) | b |  | 11768 | (-4463) | b |  | 14916 | (-2316) | a |
| Kh_r_ | m^4^·s^-1^·Mpa^-1^ |  | 6.65·10^-10^ | (2.36·10^-10^) | b |  | 7.15·10^-10^ | (3.01·10^-10^) | ab |  | 7.55·10^-10^ | (1.56·10^-10^) | a |
| HCUE | m^2^·s^-1^·Mpa^-1^ |  | 5.23·10^-14^ | (1.78·10^-14^) | b |  | 6.01·10^-14^ | (2.08·10^-14^) | a |  | 4.91·10^-14^ | (1.79·10^-14^) | b |
| CN | n |  | 19.6 | (-5.7) | b |  | 19.8 | (-6.3) | b |  | 34.5 | (-7.7) | a |
| RW | µm |  | 793 | (-266) | b |  | 764 | (-275) | b |  | 1153 | (-230) | a |

Supplementary Table S1. Mean (and standard deviation) parameters of larch at S22, larch at S19 and spruce at S19. The parameters were calculated from seven trees in each site and species for the period 1900-2017. Different letters indicate significant differences in the row, according to the Kruskal–Wallis and Dunn’s post-hoc tests. Parameters are: tree diameter at breast height (DBH), ring number (RN), cell lumen radial diameter (CLD), mean cell-wall thickness (CWT), cell lumen area (CLA), cell-wall area (CWA), cell total area (CTA), theoretical hydraulic cell conductivity (Kh_c_), relative anatomical cell wood density (CWD), total wall area in the radial file (RWA), tree-ring specific hydraulic conductivity (Kh_r_), cell number (CN), hydraulic carbon use efficiency (HCUE), and ring width (RW).

|  | Year | CLD | CWT | CLA | CWA | CTA | Kh_c_ | CWD | RWA | Kh_r_ | HCUE | CN | RW |
| --- | --- | --- | --- | --- | --- | --- | --- | --- | --- | --- | --- | --- | --- |
| Larch S22 high severity | 1908 | 1.02 | 0.88 | 1.01 | 0.88 | 0.96 | 0.99 | 0.93 | 0.64 | 0.72 | 1.12 | 0.73 | 0.70 |
|  | 1945 | 1.11 | 0.87 | 1.19 | 0.94 | 1.08 | 1.30 | 0.87 | 1.08 | 1.46 | 1.41 | 1.14 | 1.20 |
|  | 1963 | 1.13 | 0.77 | 1.19 | 0.84 | 1.02 | 1.24 | 0.82 | 0.65 | 0.89 | 1.53 | 0.75 | 0.75 |
|  | 1972 | 1.06 | 0.80 | 1.08 | 0.82 | 0.95 | 1.11 | 0.91 | 0.77 | 1.03 | 1.35 | 0.93 | 0.92 |
|  |  |  |  |  |  |  |  |  |  |  |  |  |  |
| Larch S22 low severity | 1915 | 1.00 | 1.03 | 1.03 | 1.03 | 1.02 | 1.09 | 1.02 | 1.01 | 1.09 | 1.05 | 0.99 | 1.00 |
|  | 1937 | 0.99 | 0.97 | 0.99 | 0.96 | 0.98 | 0.99 | 1.00 | 0.89 | 0.91 | 1.03 | 0.92 | 0.90 |
|  | 1954 | 0.92 | 0.91 | 0.85 | 0.85 | 0.83 | 0.79 | 1.06 | 0.67 | 0.62 | 0.92 | 0.79 | 0.68 |
|  | 1981 | 1.04 | 0.91 | 1.06 | 0.91 | 0.98 | 1.08 | 0.94 | 0.79 | 0.98 | 1.20 | 0.88 | 0.90 |
|  |  |  |  |  |  |  |  |  |  |  |  |  |  |
| Larch S19 high severity | 1908 | 1.13 | 0.69 | 1.12 | 0.76 | 0.93 | 1.07 | 0.79 | 0.36 | 0.49 | 1.43 | 0.46 | 0.44 |
|  | 1945 | 0.93 | 0.66 | 0.74 | 0.61 | 0.67 | 0.55 | 0.86 | 0.20 | 0.21 | 0.83 | 0.32 | 0.32 |
|  | 1963 | 1.10 | 0.53 | 0.99 | 0.60 | 0.79 | 0.75 | 0.66 | 0.18 | 0.23 | 1.21 | 0.28 | 0.25 |
|  | 1972 | 1.27 | 0.57 | 1.31 | 0.73 | 1.03 | 1.17 | 0.60 | 0.38 | 0.62 | 1.60 | 0.52 | 0.55 |
|  |  |  |  |  |  |  |  |  |  |  |  |  |  |
| Larch S19 low severity | 1915 | 1.08 | 0.80 | 1.08 | 0.85 | 0.97 | 1.02 | 0.87 | 0.64 | 0.78 | 1.20 | 0.73 | 0.73 |
|  | 1937 | 1.06 | 0.87 | 1.06 | 0.89 | 0.96 | 1.04 | 0.93 | 0.64 | 0.75 | 1.18 | 0.71 | 0.71 |
|  | 1954 | 1.03 | 0.93 | 1.01 | 0.96 | 0.97 | 1.01 | 0.99 | 1.13 | 1.17 | 1.06 | 1.14 | 1.09 |
|  | 1981 | 1.01 | 0.81 | 0.96 | 0.80 | 0.90 | 0.86 | 0.87 | 0.49 | 0.54 | 1.08 | 0.60 | 0.54 |

Supplementary Table S2. Ratio between mean ring anatomical parameters in the first outbreak year, and the mean value of the previous five years (reference), for larch at S19 and S22 and for high- and low-severity outbreaks. Parameters are: cell lumen radial diameter (CLD), mean cell-wall thickness (CWT), cell lumen area (CLA), cell-wall area (CWA), cell total area (CTA), theoretical hydraulic cell conductivity (Kh_c_), relative anatomical cell wood density (CWD), total wall area in the radial file (RWA), tree-ring specific hydraulic conductivity (Kh_r_), hydraulic carbon use efficiency (HCUE), cell number (CN), and ring width (RW).


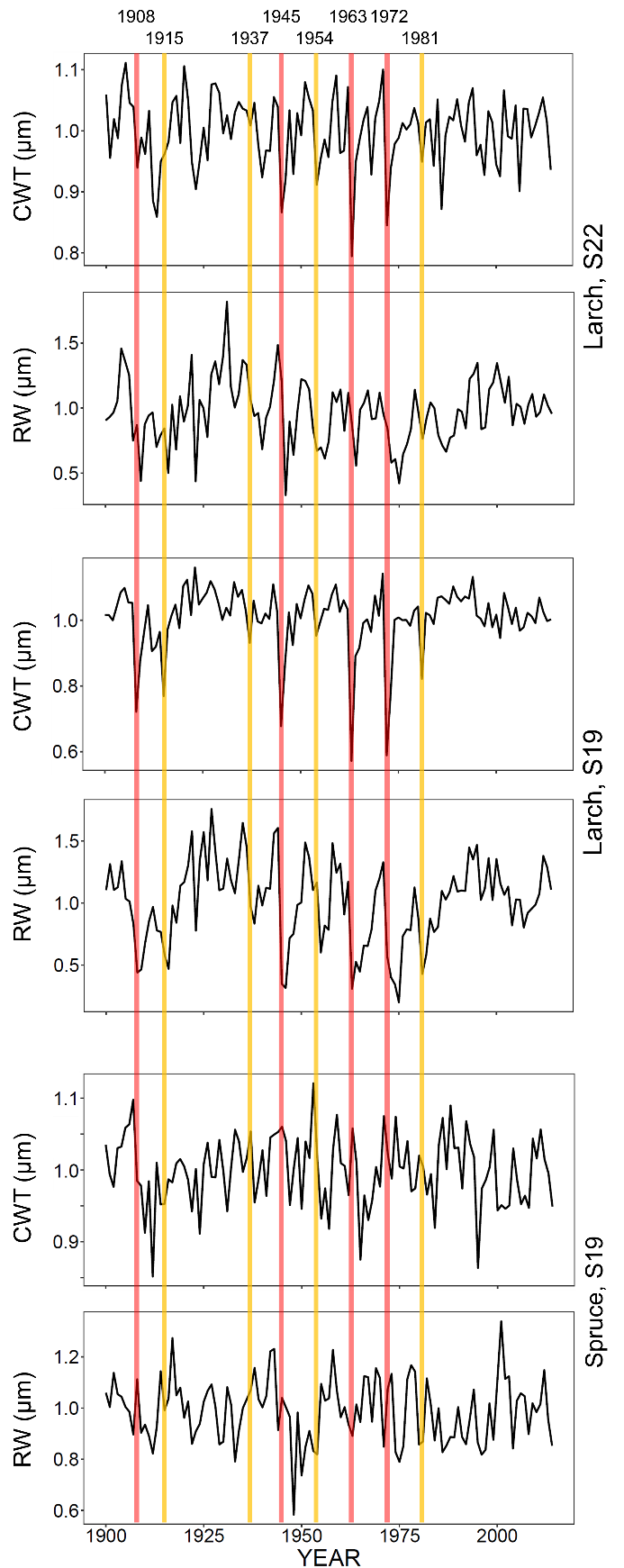


Supplementary Figure S1. Standardized chronologies of cell-wall thickness (CWT) and ring width (RW) from 1900 to 2017 for larch at S22, larch at S19 and spruce at S19. Vertical lines represent the first years of high- (in red) and low-severity (in yellow) outbreaks.


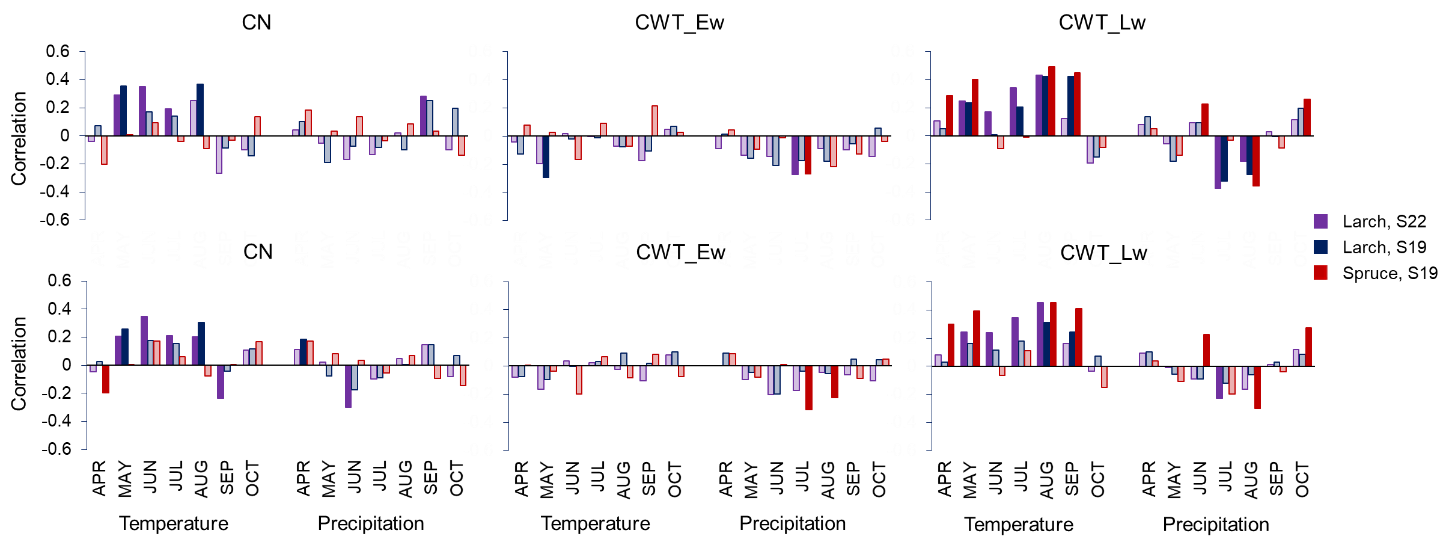


Supplementary Figure S2. Pearson’s correlations between indexed chronologies of cell number (CN) and earlywood and latewood cell-wall thickness (CWT_Ew and CWT_Lw), and monthly (April to October) temperature and precipitation (from Crans-Montana weather station, MeteoSwiss) in larch at S22, larch at S19 and spruce at S19. Correlations were calculated for the period 1931 – 2017, using chronologies excluding (three upper panels) and including (three lower panels) outbreak years. Dark colored bars indicate significant correlations (P<0.05), light colored bars indicate not significant correlations (P≥0.05). See the key for correspondence between colors and species and sites.


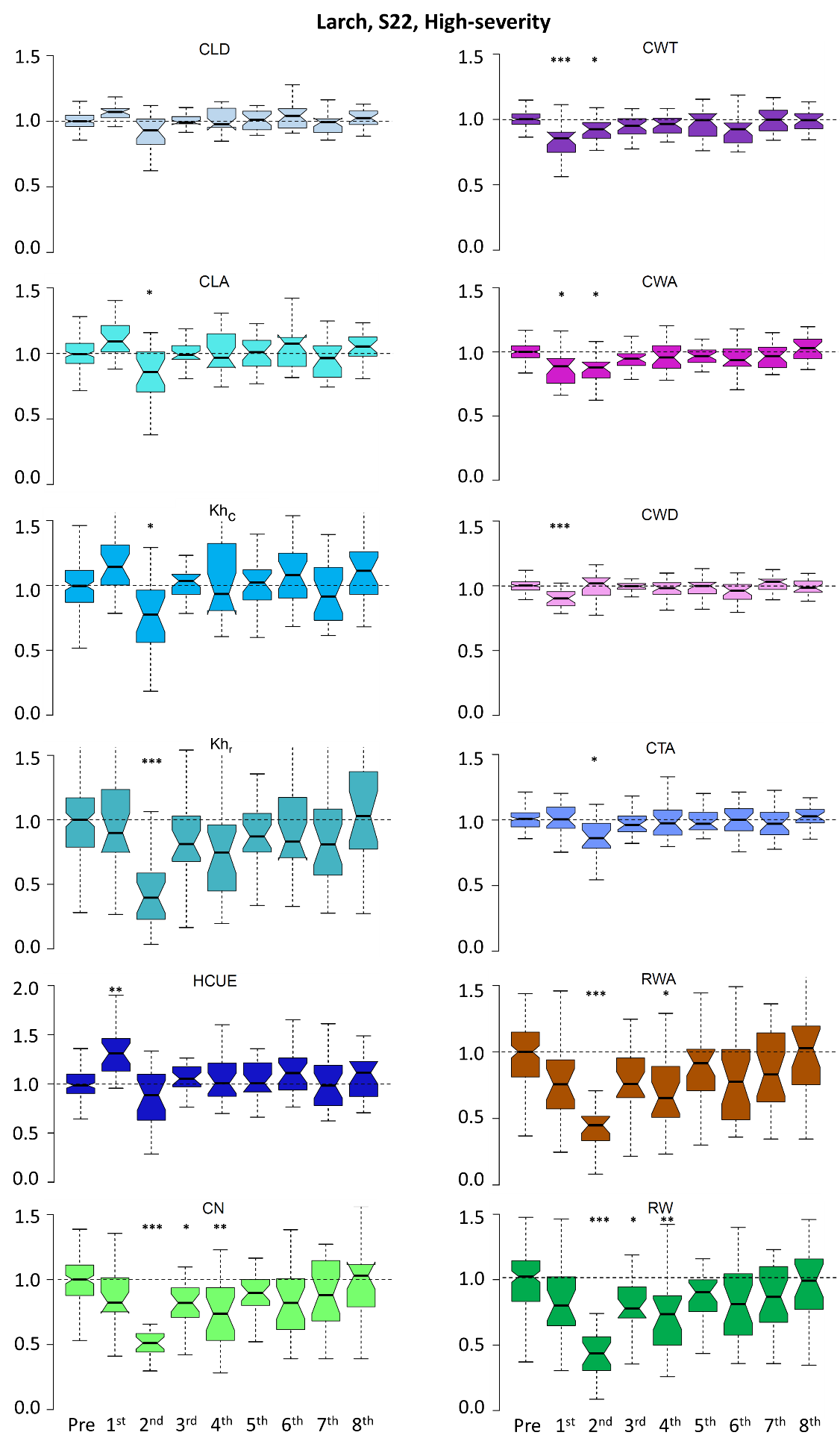


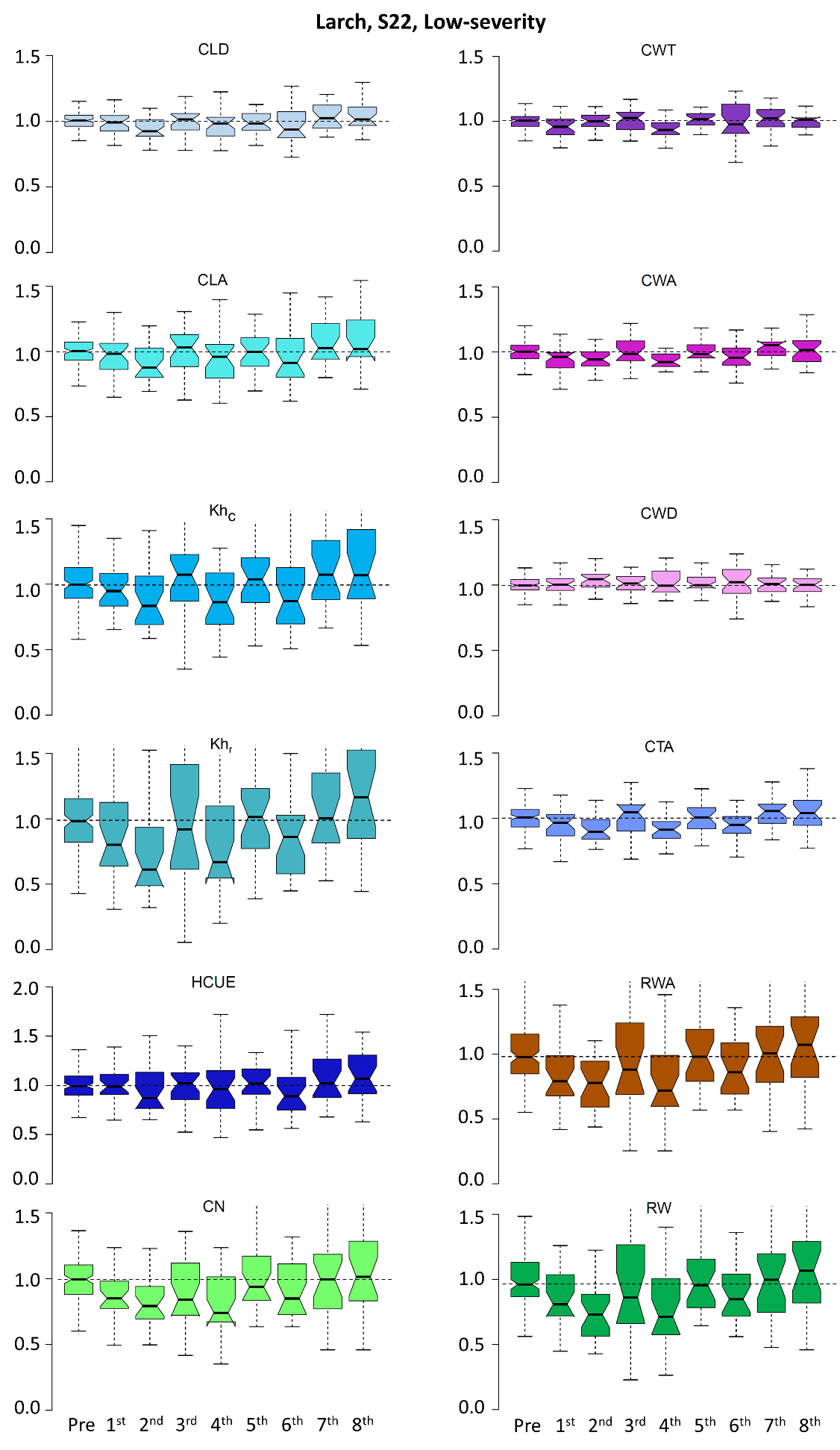


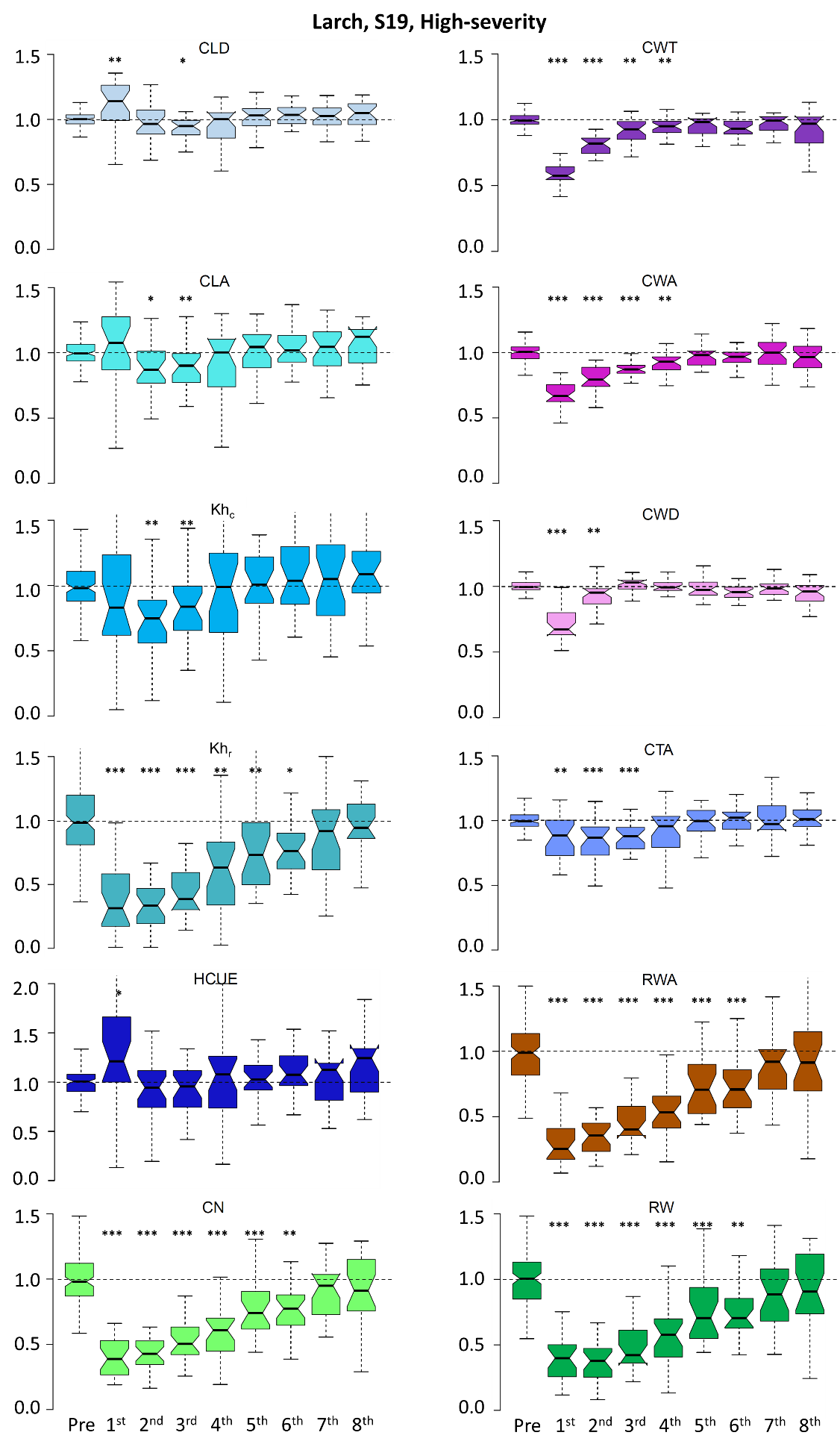


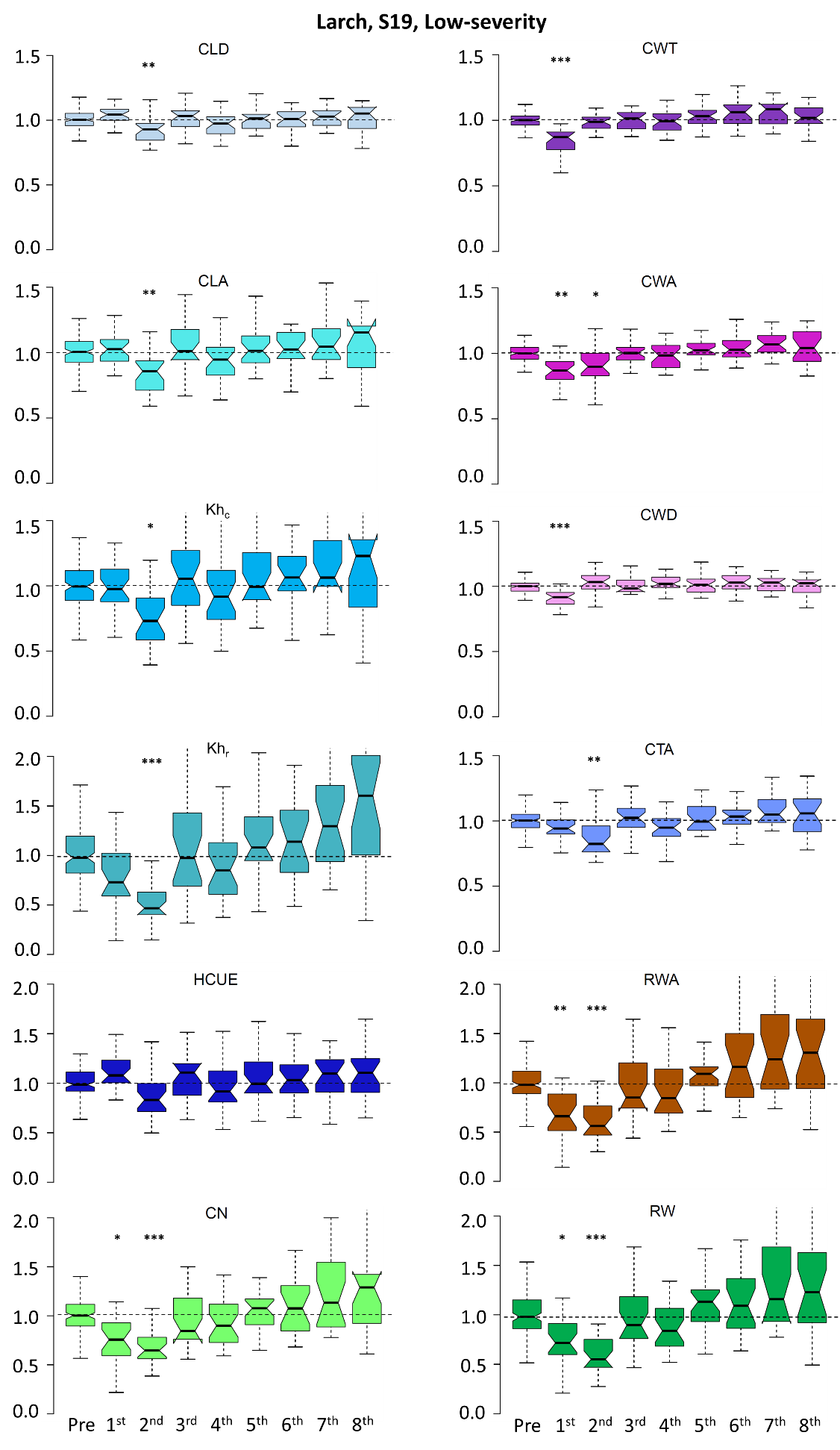


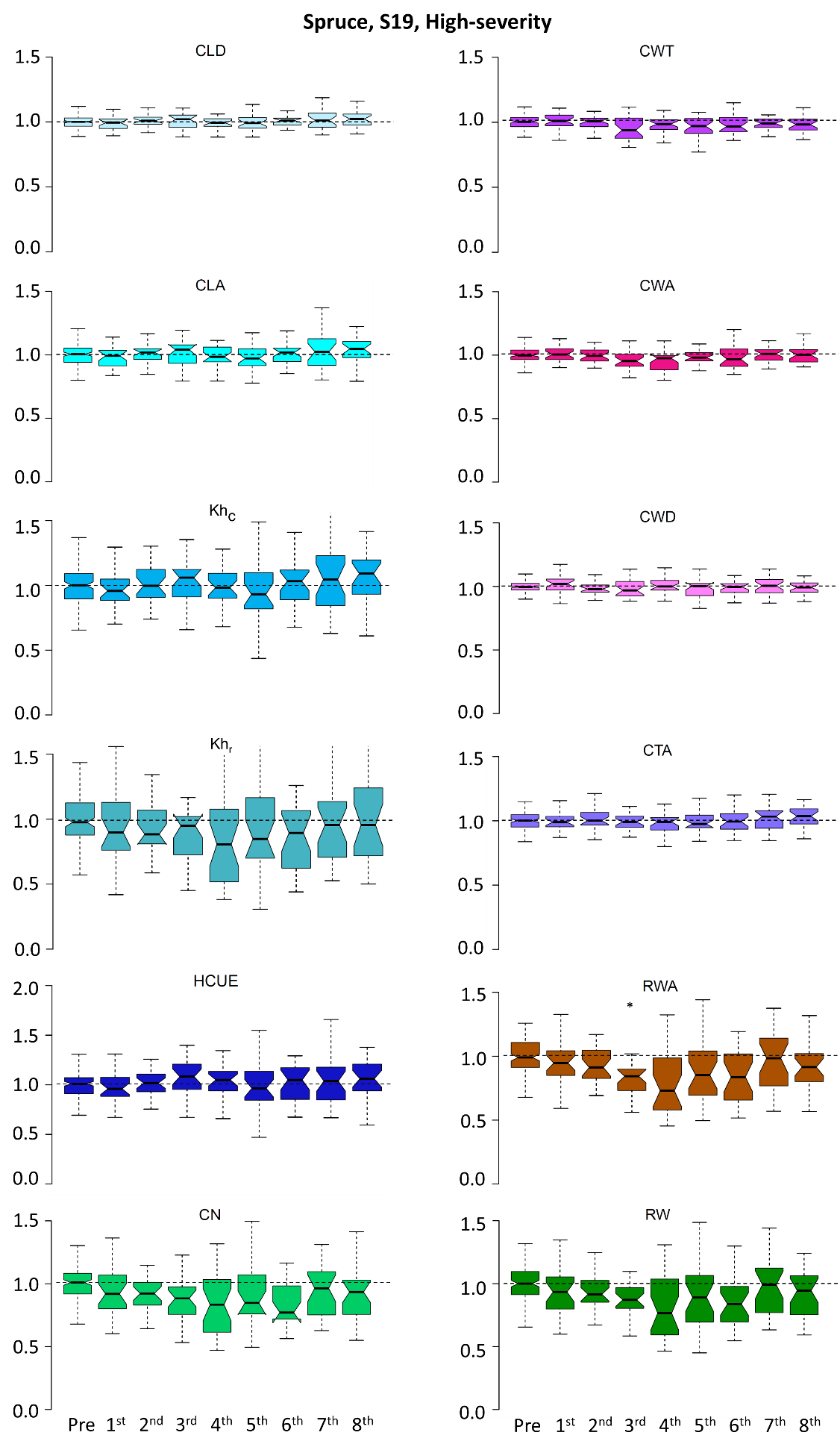


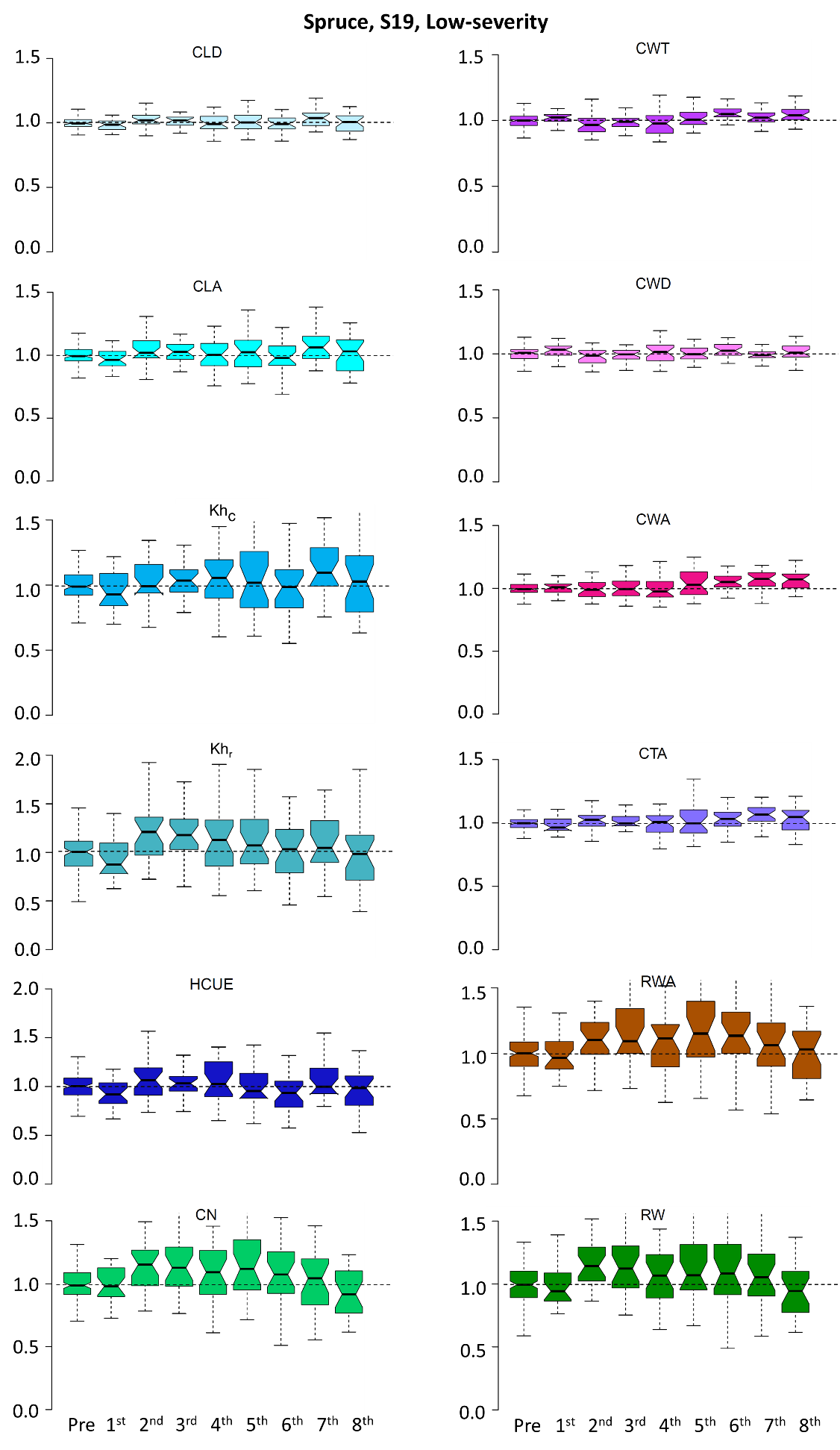


Supplementary Figure S3. Boxplots of the ratios between xylem parameters in the outbreak and following years (from 1^st^ to 8^th^) and the reference (“Pre”). Each box represents median, interquartile range (IQR), confidence interval (notches, +/- 1.58·IQR/√n), minimum and maximum deviation from the reference, i.e. the mean value during the five years before the outbreak (“Pre”). Information for the year 1908, 1945, 1963 and 1972 are merged to represent the effects of high-severity outbreaks. Information for the year 1915, 1937, 1954 and 1981 are merged to represent the effects of low-severity outbreaks. Asterisks indicate significant difference from the reference at p<0.05 (*), p<0.01 (**) and p<0.001 (***) according to Welch’s t-test.


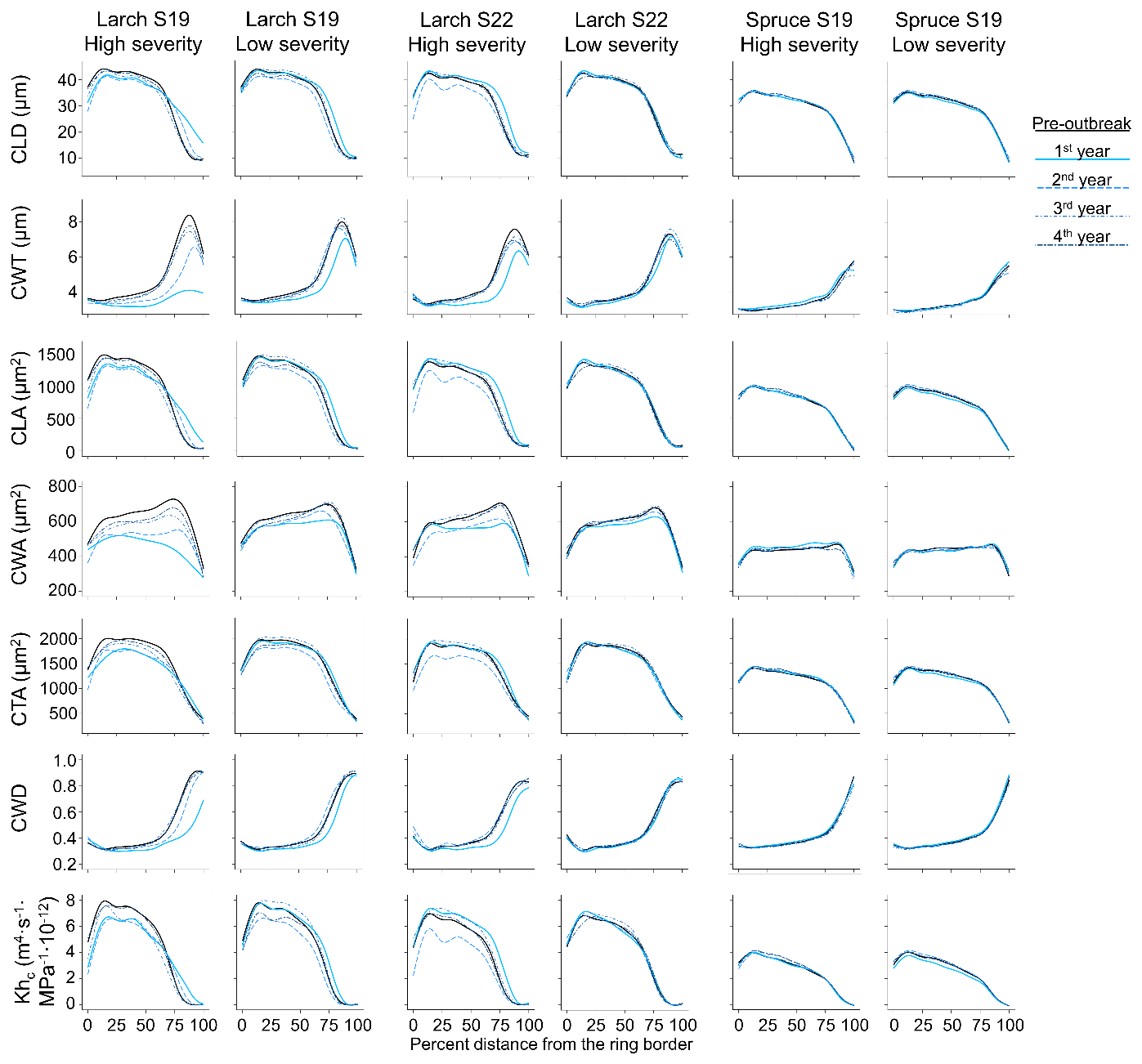


Supplementary Figure S4. Evolution of cell anatomical characteristics around the outbreak events for the four high- and low-severity outbreaks at S22 (larch) and S19 (larch and spruce). Pre = average of the five years before outbreak. 1^st^, 2^nd^, 3^rd^, and 4^th^ are the outbreak years (see the key for correspondence between years and line style). CLD = cell lumen radial diameter; CWT = cell-wall thickness; CWA = cell wall area; CLA = cell lumen area; CWA = cell-wall area; CTA = cell total area; CWD = relative anatomical cell wood density; Kh_c_ = theoretical hydraulic cell conductivity. All parameters are standardised as a percentage of distance from the ring border (intra-ring profiles).
